# National Service and the Hospitals

**Published:** 1915-06-05

**Authors:** 


### The Hospital

The Workers' Newspaper of Administrative Medicine and Institutional Life, Administration, National Insurance and Health.

No. 1511, Vol. LVIII. SATURDAY, JUNE 5, 1915. PRICE ONE PENNY

### NATIONAL SERVICE AND THE HOSPITALS.

National Service to-day means that the whole able-bodied population must come forward to serve not only in the Navy and the Army, but in any capacity in which the Government declare their services to be needed. The voluntary hospitals, and those attached to them, have long ago accepted this wide view of the meaning of National Service, and the latter have applied it with self-denial and energy to themselves and their institutions. At the outset large requests were made by both Navy and Army for bed accommodation for the wounded. The War Office demands have been increased on more than one occasion, and to-day many hospitals find themselves unable to meet these further requirements by the authorities, and at the same time to maintain the reputation and high efficiency of their establishments. Some voluntary hospital managers have refused to take payment from the Government, but these represent a small minority, whose objections rest mainly on misapprehension. Where there is sound organisation and business management, there the voluntary hospitals are obtaining payment, which, in the case of the London Hospital, represents a sum of about £4,000 a quarter. The War Office is evidently satisfied with the arrangement to obtain accommodation by payment, for they have desired several voluntary hospitals still further to increase the number of beds that they have placed at the disposal of the military authorities.

The present position is that the adequate discharge of the responsibilities of the managers of many voluntary hospitals must prevent them from further attempting to supply additional beds for the sick and wounded from the war. The accommodation already provided for the War Office by the voluntary hospitals has placed a severe strain upon the hygienic efficiency and resources of many establishments. It has naturally been the desire of the voluntary hospitals not to curtail in any way the accommodation provided for the comfort of the civilian population. In some of the larger hospitals where the efficiency in normal conditions is excellent, the authorities have consented to place additional beds in several of the wards, thus providing an increase of 33 per cent. in the bed accommodation available. Where this course has been taken it is recognised that the extra accommodation can only continue during the period of the war. It must be manifest that it would be impossible to increase the number of beds placed in every hospital ward to the extent of 33 per cent. Necessarily, every increase of the kind has had to be carefully considered by the medical staff and those responsible for the hygienic efficiency of each ward. These considerations place a limit to the bed accommodation it is possible to provide at each hospital. Many of the hospitals have garden and grass space where it might be practicable to erect temporary pavilions, but in addition to hygienic considerations it is apparent that no hospital worthy of the name could sanction the erection of such extra accommodation unless it has the staff available to work the new wards. It is useless for a voluntary hospital to offer beds when it cannot at the same time supply the services of medical men, nurses, ward maids, and other workers to make them efficient. We are glad to know that the London Hospital has set an example to the whole of the country by its managers declining to attempt to enlarge the number of its beds placed at the service of the War Office, beyond the maximum which they find it possible to work with the fullest efficiency. The managers of our voluntary hospitals are rendering magnificent service to the nation, but they cannot do and must not attempt impossibilities.

When an increased demand is made by the authorities on a voluntary hospital for still further bed accommodation two alternatives are open to its managers. One is to recognise their limitations and to follow the policy so wisely pursued by the London Hospital. This is to decline to attempt to supply more beds than they can properly equip and administer. But there are hospital authorities possessing a highly trained and most efficient staff of administrators who could, as events have shown, render much-needed service to the Government by supplementing the inadequate supply of trained and technically knowledgeable administrators from which the War Office suffers at the present time. The experience of the past few months reveals that the War Office has found it impossible promptly to accept offers of a site on which to erect temporary wards in connection with an existing hospital, because they were unable to supply the contractors immediately to erect these buildings. It has proved possible, however, for the hospital authorities immediately to find a contractor to put up forthwith the new wards on a plan prepared under the direction of the hospital authorities. The truth is, a great hospital is a most invaluable customer to the firms from which it obtains its supplies. This gives the hospital authorities a driving force not possessed by the War Office, and the best business men working our voluntary hospitals to-day, if they were to be co-ordinated to the War Office by the Government declaring their services were needed, could speedily supply energy. We hope that the wider meaning which attaches to National Service under a Coalition Government may speedily strengthen the War Office administration, at least so far as the detailed and technical aspects of further hospital equipment and hospital service are concerned. We have been consulted by civilians, men of position in the hospital world, who possess high technical qualifications and administrative gifts, who are prepared to give much time and gratuitous service in any capacity where they can be useful. There is, therefore, no justification for continuing a system whereby some of the great heads of the departments are unnecessarily and dangerously overworked. Nor is it business to permit important branches of work which urgently require to be done with dispatch to be left longer in the hands of men who have not the technical knowledge nor the driving force to put it through quickly.

